# Impact of Preseason Climate Factors on Vegetation Photosynthetic Phenology in Mid–High Latitudes of the Northern Hemisphere

**DOI:** 10.3390/plants13091254

**Published:** 2024-04-30

**Authors:** Kunlun Xiang, Qian Guo, Beibei Zhang, Jiaming Wang, Ning Jin, Zicheng Wang, Jiahui Liu, Chenggong Wang, Ziqiang Du, Liang Wang, Jie Zhao

**Affiliations:** 1 Guangdong Ecological Meteorology Center, Guangzhou 510640, China; xiangklun@mail2.sysu.edu.cn; 2 Chongqing Institute of Meteorological Sciences, Chongqing 401147, China; 3 Guangzhou Meteorological Satellite Ground Station, Guangzhou 510640, China; guoq18@lzu.edu.cn; 4 Shandong Provincial Key Laboratory of Water and Soil Conservation and Environmental Protection, College of Resources and Environment, Linyi University, Linyi 276000, China; zhangbeibei@lyu.edu.cn (B.Z.); wzc05172003@163.com (Z.W.); ljh0539ljh@163.com (J.L.); wcg02092003@163.com (C.W.); 5 College of Natural Resources and Environment, Northwest A&F University, Xianyang 712100, China; wangjiaming@nwafu.edu.cn; 6 Department of Resources and Environmental Engineering, Shanxi Institute of Energy, Jinzhong 030600, China; jinn.13b@igsnrr.ac.cn; 7 Institute of Loess Plateau, Shanxi University, Taiyuan 030006, China; duzq@sxu.edu.cn

**Keywords:** climate factor, growing season, mid–high latitudes, northern terrestrial ecosystem, vegetation photosynthetic phenology

## Abstract

During the period preceding the vegetation growing season (GS), temperature emerges as the pivotal factor determining phenology in northern terrestrial ecosystems. Despite extensive research on the impact of daily mean temperature (T_mean_) during the preseason period, the influence of diurnal temperature range (DTR) on vegetation photosynthetic phenology (i.e., the impact of the plant photosynthetic cycle on seasonal time scale) has largely been neglected. Using a long-term vegetation photosynthetic phenology dataset and historical climate data, we examine vegetation photosynthetic phenology dynamics and responses to climate change across the mid–high latitudes of the Northern Hemisphere from 2001 to 2020. Our data reveal an advancing trend in the start of the GS (SOS) by −0.15 days per year (days yr^−1^), affecting 72.1% of the studied area. This is particularly pronounced in western Canada, Alaska, eastern Asia, and latitudes north of 60°N. Conversely, the end of the GS (EOS) displays a delaying trend of 0.17 days yr^−1^, impacting 62.4% of the studied area, especially northern North America and northern Eurasia. The collective influence of an earlier SOS and a delayed EOS has resulted in the notably prolonged length of the GS (LOS) by 0.32 days yr^−1^ in the last two decades, affecting 70.9% of the studied area, with Eurasia and western North America being particularly noteworthy. Partial correlation coefficients of the SOS with preseason T_mean_, DTR, and accumulated precipitation exhibited negative values in 98.4%, 93.0%, and 39.2% of the study area, respectively. However, there were distinct regional variations in the influence of climate factors on the EOS. The partial correlation coefficients of the EOS with preseason T_mean_, DTR, and precipitation were positive in 58.6%, 50.1%, and 36.3% of the region, respectively. Our findings unveil the intricate mechanisms influencing vegetation photosynthetic phenology, holding crucial significance in understanding the dynamics of carbon sequestration within terrestrial ecosystems amidst climate change.

## 1. Introduction

Vegetation phenology encompasses various natural cyclic processes, such as budding, ripening, and dormancy [1,2]. These phenomena are intricately tied to seasonal variations in temperature, precipitation, soil moisture, sunshine duration, and other environmental factors [3]. As a sensitive bioindicator, vegetation phenology responds to climate change, playing a crucial role in regulating carbon cycling within terrestrial ecosystems [4,5]. Of particular importance are the start and end of the vegetation growing season (GS), which significantly impact the productivity and carbon flux of natural ecosystems, creating feedback loops in the context of global climate change [6,7]. Consequently, investigating vegetation phenological changes in response to climate change holds promise for shedding new insight into research on the carbon cycle and regional or global land–atmosphere interactions.

Several recent studies conducted in mid–high latitudes of the Northern Hemisphere (MH-NH) have observed an early start of the GS (referred to as SOS) and a later end of the GS (referred to as EOS) [8,9]. However, the focal point of most of these studies is the structural changes exhibited by plants. For instance, such studies often utilize indicators of greenness, such as the enhanced vegetation index and the normalized difference vegetation index, to depict the advancement of leaf growth [10]. While these indicators adeptly capture fluctuations in chlorophyll levels and structural modifications, they tend to disregard shifts in vegetation photosynthesis, particularly in the context of evergreen plant species. Recent research has revealed that approaches reliant on vegetation indices struggle to accurately represent photosynthesis changes in certain types of vegetation, such as evergreen forests [11]. This discrepancy arises from occasional disparities between greenness and photosynthesis. The imprecise assessment of phenology based on vegetation greenness can consequently introduce notable uncertainties when estimating vegetation productivity and carbon sequestration.

Vegetation photosynthetic phenology, delving into the manifestations of plant physiological activities, diverges from the conventional greenness-based phenological shifts that encompass structural changes like bud break and leaf coloring [6]. Monitoring photosynthetic phenology on a broader scale can yield crucial insights into the carbon cycle and contribute to comprehending the forces propelling carbon dynamics. The delineation of photosynthetic phenology is rooted in the extraction of photosynthesis transition dates from the time series of vegetation gross primary productivity (GPP) [6]. At present, GPP can be obtained through two primary methods: eddy covariance flux towers, which provide observations on the ecosystem scale, and through modeling or remote sensing on regional and global scales [12]. The eddy covariance technique, widely recognized as an exceptionally precise observational approach, has furnished long-term GPP estimates for over two decades [13]. However, limitations in the spatial distribution of these observations result in the underrepresentation of specific critical areas [12]. Solar-Induced Chlorophyll Fluorescence (SIF), originating from the emission of plant chlorophyll molecules following the absorption of photosynthetically active radiation, is widely recognized as a potent tool for diagnosing terrestrial photosynthesis [11,14]. Therefore, the recently published dataset of photosynthetic phenological indicators, generated from GPP products derived from satellite-based SIF data, opens up new opportunities for large-scale studies on the photosynthetic characteristics of plants [6]. 

Alterations in plant phenology can be notably linked to climate change, as it is predominantly influenced by preseason temperature and precipitation patterns [15,16]. Additional climatic elements, such as atmospheric carbon dioxide concentration, sunshine duration, and occurrences of extreme weather events, also play a notable role [17]. The early onset of the SOS and the delayed EOS has resulted in an extended photosynthetic cycle in plants, which contributes to the carbon sequestration effect and has the potential to influence the balance of surface energy [18]. The impact of preseason accumulated precipitation and mean temperature (T_mean_) on vegetation phenology has been extensively studied; however, plants do not only perceive variations in ambient temperature, but also exhibit sensitivity to a range of temperature fluctuations and extreme temperatures [19]. To date, there has been insufficient exploration of the vegetation phenology response to changes in preseason diurnal temperature range (DTR). Previous research in plant physiology has suggested several significant associations: (1) DTR is correlated with photoperiod [20], which can notably influence the circadian rhythm of plants. (2) Preseason DTR may control seed dormancy and consequently influence germination rates [21]. (3) Preseason DTR can impact net photosynthate assimilation and various aspects of plant development [22]. Therefore, changes in DTR resulting from asymmetric diurnal warming offer an additional plausible mechanism shedding light on shifts in vegetation phenology.

Given that DTR operates independently of T_mean_ and consolidates data related to both T_max_ and T_min_, we propose that preseason DTR serves as a valuable ecological metric for elucidating the correlations between interannual shifts in vegetation phenology and asymmetric diurnal temperature variations. Moreover, we anticipate that the impact of preseason DTR on vegetation phenology differs from that of preseason T_mean_. The primary objectives of this research were to (1) uncover the spatiotemporal variations in vegetation photosynthetic phenology over the period 2001–2020; and (2) investigate the impact of different climate factors (i.e., T_mean_, DTR, and precipitation) on the variations in vegetation photosynthetic phenology.

## 2. Results

### 2.1. Vegetation Photosynthetic Phenology and Its Temporal Variations

#### 2.1.1. Start of the Growing Season

**Figure 1:**
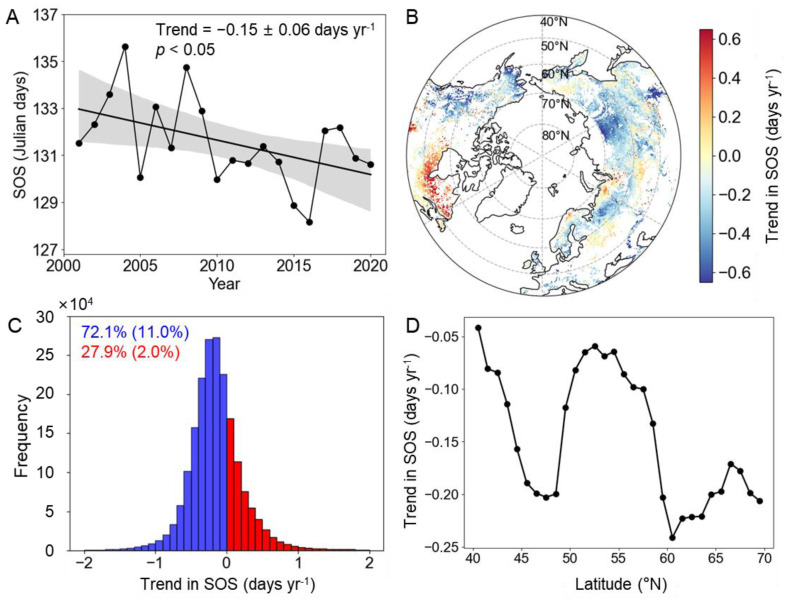
Changes in the SOS in the MH-NH from 2001 to 2020. (**A**) The overall trends in SOS variations. (**B**) Spatial distribution of linear trends in SOS variations. (**C**) The frequency of advanced and delayed trends in SOS variations. (**D**) The changes in the average slope of the SOS with latitude. Note: The shading in plot (**A**) represents 95% prediction intervals. The numbers in blue and red in plot (**C**) represent the percentages of advance and delay, respectively (*p* < 0.05).

The mean dates of the SOS in the MH-NH displayed significant advancement, with rates of −0.15 ± 0.06 days yr^−1^ from 2001 to 2020 (Figure 1A,C). Spatially, the advancement of the SOS was widespread, affecting over 72% of the region. Notably, 22.1% of pixels exhibited significant advances (*p* < 0.05) (Figure 1B,C). An advanced SOS was mainly observed in western Canada, Alaska, eastern Asia, and latitudes above 60°N. Conversely, the northeast of North Ameria and part of central Eurasia experienced a later SOS during the same period. Regions characterized by significant SOS delays constituted only around 2% of the entire study area (Figure 1B). Regarding latitude patterns, the region between 60°N and 65°N had the highest rate of advanced SOS signals, followed by 45°N to 50°N, while the regions between 50°N and 55°N and close to 40°N had lower rates of advanced SOS signals (Figure 1D).

#### 2.1.2. End of the Growing Season

**Figure 2:**
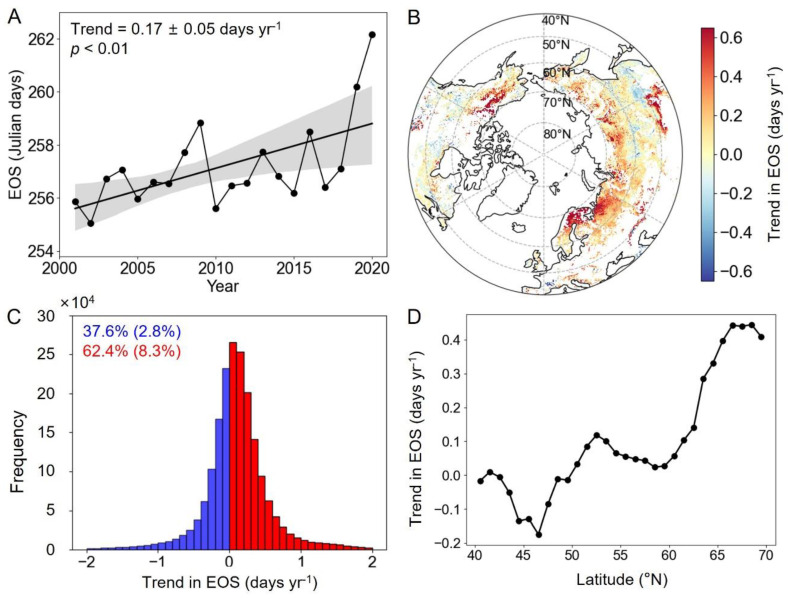
Changes in the EOS in the MH-NH from 2001 to 2020. (**A**) The overall trends in EOS variations. (**B**) Spatial distribution of linear trends in EOS variations. (**C**) The frequency of advanced and delayed trends in EOS variations. (**D**) The changes in the average slope of the EOS with latitude. Note: The shading in plot (**A**) represents 95% prediction intervals. The numbers in blue and red in plot (**C**) represent the percentages of advance and delay, respectively (*p* < 0.05).

In general, the mean dates of the EOS in the MH-NH show a delayed trend from 2001 to 2020 (0.17 ± 0.06 days yr^−1^) (Figure 2A,C). The EOS occurred later in 62.4% of the region from 2001 to 2020, with 8.3% of pixels having a significant delay trend (*p* < 0.05) (Figure 2B,C). A delayed EOS was mainly observed in northwestern Canada, northern Europe, and northern Asia (Figure 2B). Conversely, about 37.6% of the study area displayed an advanced EOS, with 2.8% of pixels having a significant advance trend (Figure 2B). The slope of the EOS exhibits a pronounced latitudinal pattern (Figure 2D). The EOS has an advancing trend south of 50°N, while in regions north of 50°N, the rate of EOS delay increases as one moves further north (Figure 2D).

#### 2.1.3. Length of the Growing Season

**Figure 3:**
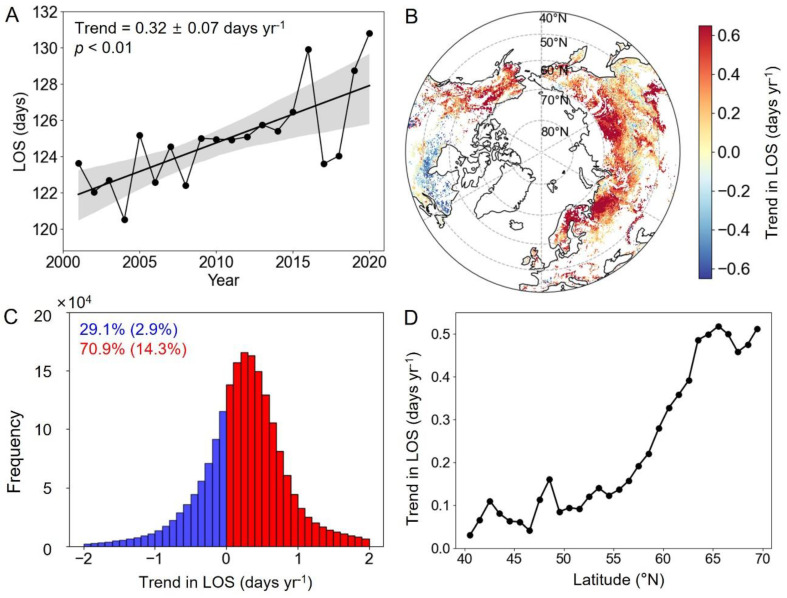
Changes in the LOS in the MH-NH from 2001 to 2020. (**A**) The overall trends in LOS variations. (**B**) Spatial distribution of linear trends in LOS variations. (**C**) The frequency of extending and shortening trends in LOS variations. (**D**) The changes in the average slope of the LOS with latitude. Note: The shading in plot (**A**) represents 95% prediction intervals. The numbers in blue and red in plot (**C**) represent the percentages of advance and delay, respectively (*p*  <  0.05).

The LOS in the MH-NH from 2001 to 2020 significantly extended by 0.32 ± 0.07 days yr^−1^ (Figure 3A,C). The spatial distribution of the LOS trends over the past two decades is illustrated in Figure 3B. From 2001 to 2020, 70.9% of northern terrestrial ecosystem pixels showed an extension trend in LOS, of which 14.3% are significant (*p* < 0.05). The longest extensions were generally located in Eurasia, Alaska, western Canada, and the western United States. Conversely, parts of northeastern North America experienced a shortened LOS during 2001 to 2020. The area with a shortened LOS accounts for 2.9% of the total study region (Figure 3B). As with EOS, the temporal trends in LOS display a notable latitudinal pattern, with the rate of LOS extension increasing as one moves further north (Figure 3D).

### 2.2. Relationships between Vegetation Photosynthetic Phenology and Preseason Climate Factors

#### 2.2.1. Relationships between SOS and Preseason Climate Factors

**Figure 4:**
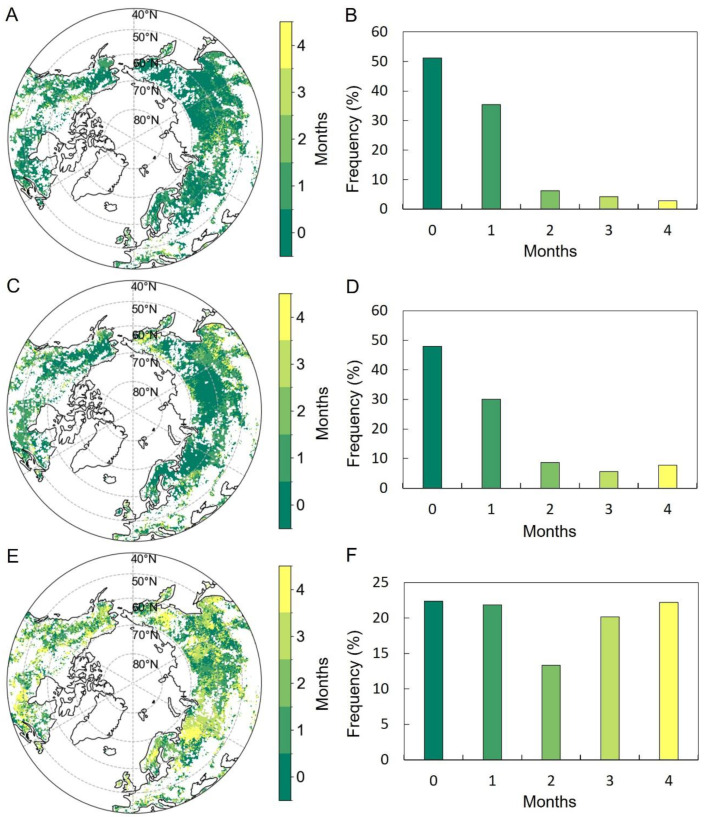
Spatial distributions of the preseason length for the partial correlation analysis of the SOS and T_mean_ (**A**), DTR (**C**), and accumulated precipitation (**E**). Uncolored pixels were excluded. Plots (**B**,**D**,**F**) depict the frequency distributions of preseason lengths corresponding to their respective variables (**A**,**C**,**E**).

The SOS preseason for T_mean_ was 0 (i.e., same month as SOS) to 1 month long over 86.6% of the MH-NH when accumulated precipitation for the corresponding period was controlled for (Figure 4A,B). Regions experiencing an extended preseason for T_mean_ (>1 month) were primarily situated in northeast China, central Russia, northwest Canada, the central United States, and southern Europe (Figure 4A). The preseason for DTR was similar to T_mean_, with a length of 0 to 1 month over 78.1% of the mid–high latitudes of the NN (Figure 4C,D). In regions such as western Canada, southeastern Europe, and northeastern Eurasia, DTR exhibited a prolonged preseason exceeding one month (Figure 4C). The preseason for accumulated precipitation exceeded that of temperature factors, spanning a duration of 1 to 4 months across 77.6% of the study area (Figure 4E,F). Preseason periods lasting 0 to 1 month were predominantly found in eastern Europe and central Eurasia, while those exceeding two months were primarily situated north of 60°N (Figure 4E).

**Figure 5:**
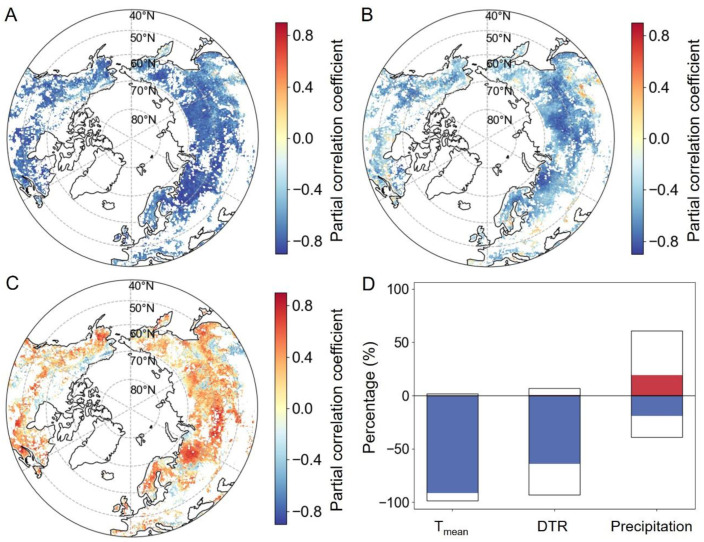
Spatial distributions of the partial correlation coefficients between preseason climate factors and the SOS. Plots (**A**–**C**) represent T_mean_, DTR, and accumulated precipitation, respectively, while plot (**D**) displays the percentages of these coefficients. The portion of the bars above the zero line in plot (**D**) indicates the percentage of positive correlation, while the portion below it indicates the percentage of negative correlation. The red and blue in plot (**D**) signify significant positive and negative correlations, respectively (*p* < 0.05).

The partial correlations between T_mean_ and SOS were predominantly negative (98.4%, Figure 5A,D), with statistically significant negative correlations observed in 75.7% of the study area (Figure 5D). Regions where partial correlations lacked statistical significance were primarily observed in the northern United States, Kazakhstan, and Mongolia. Similarly, the partial correlations between preseason DTR and SOS exhibited negativity across 93.0% of the study area, as depicted in Figure 5B,D, with statistical significance observed in 48.6% of the region (Figure 5D). Widespread significantly negative partial correlations were observed in Russia, Mongolia, northern Europe, the northern United States, and Canada. In contrast, significant positive partial correlations were observed in central Canada and southern Sweden, encompassing only 0.5% of the total area (Figure 5B,D). In addition, the partial correlation coefficients between accumulated precipitation and SOS were positive in most regions (60.8%), and significantly positive in 19.3% of the study area (Figure 5D). Regions with significantly positive partial correlations were primarily located in Russia, northeastern and western North America, and western Europe. Partial correlations between accumulated precipitation and SOS were significantly negative in southeast Europe, eastern Inner Mongolia, and in North America north of 65°N (3.6% of the total area).

#### 2.2.2. Relationships between EOS and Preseason Climate Factors

**Figure 6:**
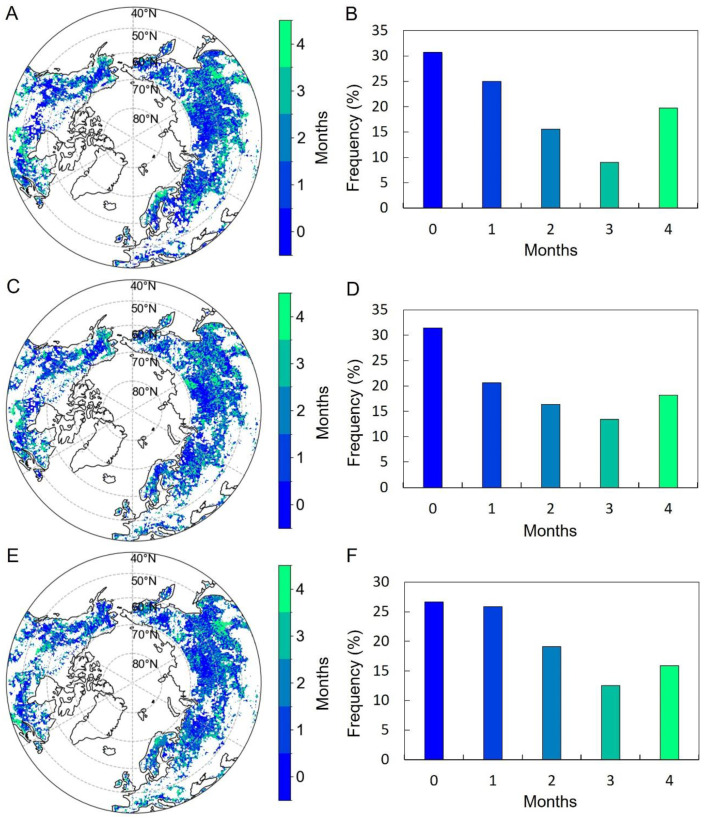
Spatial distributions of the preseason length for the partial correlation analysis of the EOS and T_mean_ (**A**), DTR (**C**), and accumulated precipitation (**E**). Uncolored pixels were excluded. Plots (**B**,**D**,**F**) depict the frequency distributions of preseason lengths corresponding to their respective variables (**A**,**C**,**E**).

The EOS preseason for T_mean_ was 0 (i.e., same month as EOS) to 1 month long over only 55.6% of the study area when the corresponding precipitation was controlled for (Figure 6A,B). Regions exhibiting a longer preseason for T_mean_ (more than 1 month) covered nearly half of the MH-NH, and were primarily concentrated in central Russia, central Europe, and the Midwest of Canada (Figure 6A). The preseason for DTR was similar to T_mean_, with a length of 0 to 1 month over about half (52.0%) of the study area (Figure 6C,D). Regions with an extended preseason for DTR (>1 month) were primarily found in southern Europe, western Canada, and northeast Eurasia (Figure 6C). The preseason for precipitation with a length of 0 to 1 month was over about half (52.5%) of the MH-NH (Figure 6E,F), mainly northeast North America, eastern Europe, and central Eurasia (Figure 6E).

**Figure 7:**
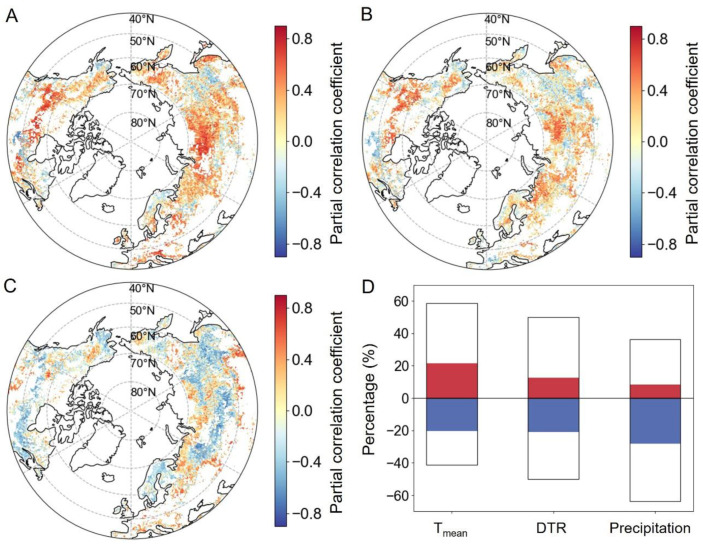
Spatial distributions of the partial correlation coefficients between preseason climate factors and the EOS. Plots (**A**–**C**) represent T_mean_, DTR, and accumulated precipitation, respectively, while plot (**D**) displays the percentages of these coefficients. The portion of the bars above the zero line in plot (**D**) indicates the percentage of positive correlation, while the portion below it indicates the percentage of negative correlation. The red and blue in plot (**D**) signify significant positive and negative correlations, respectively (*p* < 0.05).

In general, a positive correlation with T_mean_ was observed in 58.6% of the EOS pixels across the study region, as depicted in Figure 7A,D. These pixels were predominantly situated in eastern Europe, eastern North America, western North America, and Russia, with 21.4% of them passing the significance test (*p* < 0.05). Conversely, negative correlations between EOS and T_mean_ were identified primarily in the north–central United States and southeastern Russia. Furthermore, a positive relationship was observed between EOS and DTR in 50.1% of the study area, primarily in eastern Europe, central and western Asia, and North America south of 60°N, with a significance rate accounting for 12.5% of the area (*p* < 0.05) (Figure 7B,D). EOS had a negative correlation with DTR in about 49.9% of the mid–high latitudes of the NN, with 5.4% of the pixels being significant (Figure 7D). Figure 7C presents the partial correlations between EOS and preseason accumulated precipitation. There were larger areas showing negative correlations (63.7%) between EOS and precipitation than those with positive correlations (36.3%) in the study area (Figure 7D). Regions exhibiting significantly negative partial correlations were predominantly located in western Alaska, southwestern Canada, eastern Canada, northeastern United States, central Russia, and northern Europe. Pixels exhibiting significantly positive partial correlations between the EOS and preseason accumulated precipitation were predominantly located in the central United States, northern Russia, and regions of Eurasia located south of 50°N (Figure 7C).

## 3. Materials and Methods

### 3.1. Data Sources

Information regarding the annual vegetation photosynthetic phenology in the MH-NH for the period of 2001–2020 was obtained from the latest published vegetation photosynthetic phenology dataset [6]. This dataset has a spatial resolution of 0.05 × 0.05° and uses the latest GPP product based on SIF [6]. Its construction utilized a combination of smooth splines and multi-point detection techniques to extract phenological indicators such as the SOS, EOS, and the length of the GS (LOS) from terrestrial ecosystems situated at latitudes north of 30°N. The SOS and EOS dates in the dataset for each phenological cycle are determined by amplitude thresholds. Here, the 25% threshold was selected for the analysis of phenological changes and the response of vegetation photosynthetic phenology to climate factors.

The historical monthly T_max_, T_min_, and precipitation data, with a spatial resolution of 0.04° × 0.04°, spanning the period from 2000 to 2020, were acquired from the WorldClim historical monthly weather dataset (https://worldclim.org/data/monthlywth.html (accessed on 29 April 2024)) [23]. This dataset, generated through angular distance weighting interpolation, has been extensively utilized in investigations exploring the connection between vegetation activity and climate change [24]. T_mean_ is calculated as the mean of T_max_ and T_min_, while DTR is given by T_max_ − T_min_. The aforementioned datasets were further resampled to 0.05° × 0.05° by means of the nearest neighbor method to align with the resolution of the vegetation photosynthetic phenology data.

### 3.2. Spatiotemporal Variations in Vegetation Photosynthetic Phenology

The day of year (DOY) represents the sequential numbering of days, commencing with day 1 on January 1st. To investigate the spatial and temporal trends of SOS and EOS over the MH-NH, we initiated the analysis by excluding abnormal SOS values (those outside the range SOS > DOY 0 and SOS ≤ DOY 180) and abnormal EOS values (those outside the range EOS > DOY 180). Subsequently, we calculated the spatially averaged phenological metrics for vegetation photosynthesis across the MH-NH for each year. Next, we employed simple linear regression methods to calculate the temporal trends for the overall vegetation photosynthetic phenology metrics [24].

We performed this analysis using phenological metrics with 25% thresholds within the study area. The regression coefficient indicates the interannual variation in vegetation photosynthetic phenology metrics from 2001 to 2020, expressed as days per year (days yr^−1^). Furthermore, we applied the same methodology spatially, enabling us to determine the slope and *p*-value for each individual pixel over the MH-NH.

### 3.3. Relationships between Preseason Climate Factors and Vegetation Photosynthetic Phenology Metrics

We conducted a partial correlation analysis to explore the relationship between vegetation photosynthetic phenology metrics with 25% thresholds and various climatic factors. In our study, it was possible for multicollinearity to be present among several climate variables, such as T_mean_, DTR, and precipitation. To mitigate the influence of these confounding variables and obtain a clearer understanding of the relationship between individual climate factors and vegetation photosynthetic phenology metrics, we employed first-order partial correlation analysis (Equation (1)), as previously employed in studies by Du et al. (2022) [1] and Huang et al. (2020) [21].

(1) $$r_{xy\cdot z}=\frac{r_{xy}-r_{xz}r_{yz}}{\sqrt{1-r_{xz}^{2}}\sqrt{1-r_{yz}^{2}}}$$

where *r_xy__·__z_* is the first-order partial correlation coefficient between *x* and *y* after removing the influence of variable *z*. *r_xy_*, *r_xz_*, and *r_yz_* are the correlation coefficients between the two variables.

The formula for calculating the correlation coefficient is as follows:

(2) $$r_{xy}=\frac{\sum_{i=1}^{n}\left(x_{i}-\bar{x}\right)\left(y_{i}-\bar{y}\right)}{\sqrt{\sum_{i=1}^{n}{\left(x_{i}-\bar{x}\right)}^{2}\sum_{i=1}^{n}{\left(y_{i}-\bar{y}\right)}^{2}}}$$

where *r_xy_* is the correlation coefficient between variables *x* and *y*. *x_i_* and *y_i_* are individual data points for *x* and *y*. $\bar{x}$ and $\bar{y}$ are the means of *x* and *y*, respectively.

To determine the length of the preseason during which climatic factors exert the greatest influence on vegetation photosynthetic phenology metrics, we calculated the partial correlation coefficients between the vegetation photosynthetic phenology metrics and climate factors at 0 to 4 months before the multi-year mean vegetation photosynthetic phenology metrics in the MH-NH for the period 2001–2020. We selected the time period with the maximum absolute value of the partial correlation coefficients as the most relevant preseason timeframe. Additionally, we calculated *p*-values for each pixel to assess the statistical significance of our findings. This study also utilized the partial correlation analysis method to examine the relationships between vegetation phenological indicators based on 10% thresholds and various climate factors (Figures S1–S4). As the results closely resemble those based on 25% thresholds, they are not presented in the main text.

## 4. Discussion

### 4.1. Temporal and Spatial Trends in Vegetation Photosynthetic Phenology

Utilizing long-term photosynthetic phenology data, we have identified a significant advancement in the SOS within northern terrestrial ecosystems. This observation is based on a comprehensive analysis of photosynthetic phenology, aligning with prior studies that employed greenness indicators to investigate a similar trend [25,26]. In particular, Chen et al. (2022) found a slightly different magnitude, noting a 2.08-day average advancement in the SOS over the NH during 2001–2018. Despite the slight variations in magnitude, the overall trend of an earlier SOS is consistent with the present study. The disparities among these studies may stem from differences in methodologies for determining phenological dates and variations in the time intervals covered. However, it is noteworthy that, at a regional level, a distinct trend of a delayed SOS has been evident across the majority of the regions in northeastern North America in recent decades. This observation aligns with previous research findings, indicating a gradual weakening of the carbon sink capacity in these regions [25].

We found that the EOS over the MH-NN exhibited a significant delayed trend, again consistent with previous studies [27]. There was a distinct latitudinal pattern observed in the temporal trends of the EOS. In contrast to certain prior studies [28], our findings indicate a delay in the EOS at high latitudes (>50°N) and an advancement at mid-latitudes (40–50°N). This pattern suggests a progressive strengthening of carbon sink capacity at high latitudes, while indicating a decline in the mid-latitudes under the influence of climate warming. Differences in EOS slopes between this study and previous ones may result from variations in satellite observations, data preprocessing methodologies, and the inherent uncertainties associated with these factors [29].

### 4.2. Response of SOS to Climate Change

This study revealed that warming expedited the SOS for vegetation across the majority of northern terrestrial ecosystems. Two potential mechanisms underlie these observations. Firstly, plants in mid–high latitudes of the NH require a critical level of forcing temperature, such as growing degree days, to initiate spring leaf onset [30]. The cumulative sum of daily T_mean_ above a fixed threshold value, known as growing degree days, serves as a common proxy for the heat accumulation required for leaf unfolding [31]. Consequently, the increased T_mean_ in spring more effectively satisfies the growing degree days requirement that triggers leaf onset. Secondly, the rise in T_mean_ can influence soil temperature and moisture availability—both crucial factors for plant growth. Elevated temperatures may hasten soil warming, improving water availability for plants and thereby fostering their growth.

Over the past 50 years, temperature data suggest that nighttime warming in northern land areas has outpaced daytime warming [32], leading to a diminished trend in DTR. This asymmetrical warming has been confirmed to impact vegetation productivity [33], and our research further suggests that changes in DTR can also have a significant effect on the SOS of vegetation. Primarily, a significant negative correlation between DTR and the SOS of vegetation exists across the vast majority of regions (Figure 5). The impact of preseason DTR on the SOS can be attributed to effects stemming from T_max_ and T_min_. Firstly, when daytime warming in spring is slower than nighttime warming, it is more difficult for environmental conditions to reach the critical temperature needed to activate the SOS, thus leading to the delay of the SOS [30]. Secondly, the accelerated warming of T_min_ prevents some plant species from meeting their chilling requirements [34]. In this case, a decrease in DTR may lead to a delay in the spring phenology of these species. Thirdly, the faster warming of T_min_, leading to a reduction in DTR, may result in a negative SOS-DTR correlation. In high latitudes, this scenario arises when the warming impact of clouds in winter exceeds terrestrial radiative cooling, resulting in elevated nighttime temperatures [35]. Nevertheless, a mild preseason can trigger early plant development, rendering plants more vulnerable to late frosts [36]. In a warmer climate, escalating spring frost damage can consequently lead to a delayed spring phenology [37,38].

The impact of precipitation on the SOS for vegetation is intricate, and under diverse geographical and meteorological conditions, this impact can manifest in various ways [39]. Firstly, ample precipitation is pivotal for ensuring sufficient water supply in the soil. A moderate moisture level aids nutrient absorption by plants, fostering growth and positively impacting the advancement of the vegetation SOS [40]. Secondly, it is noteworthy that increased preseason precipitation, with its accompanying deficient sunshine intensity and duration, may lead to lower temperatures, potentially resulting in a delayed SOS [41]. Lastly, certain plants employ adaptive mechanisms to cope with varying moisture conditions. Some plants adjust root structures, engage in water storage, or employ other physiological adaptations to navigate different precipitation levels, consequently influencing their SOS. The intricate impact of precipitation on the SOS of vegetation gives rise to distinct spatial patterns, as evidenced in the findings of this study.

### 4.3. Response of EOS to Climate Change

We found that that across most regions, especially in colder areas such as high latitudes, a warming preseason climate is associated with a postponement of the EOS, aligning with previous studies that drew upon field experiments and satellite data [3,42,43]. The positive effect of preseason climate warming on the EOS is likely attributed to the augmented activity of photosynthetic enzymes driven by warming [44], reduced chlorophyll degradation speed [45], diminished frost exposure likelihood in autumn, and increased potential for growth and photosynthetic consumption [44]. In contrast, negative temperature–EOS correlations were detected in arid and semi-arid regions, such as central Eurasia and the north–central United States. This effect could be linked to warmer autumns substantially decreasing water availability in dry locales [46], adversely impacting plant growth and photosynthesis [47] and increasing chlorophyll degradation and plant mortality risks [48,49], consequently leading to an earlier EOS.

Unlike the SOS, DTR exhibits a distinct spatial pattern in its impact on the EOS, indicating that the response mechanism of vegetation dormancy to DTR is a complex physiological and ecological process (Figure 7). Some aspects of the response mechanism of vegetation dormancy to DTR are as follows: Firstly, a smaller DTR is induced by a rapid increase in T_min_. A swift rise in T_min_ helps prevent plants from experiencing cold stress [21], thereby delaying the onset of dormancy. Secondly, the rapid increase in T_min_ influences soil temperature [50], keeping it relatively high, which, in turn, sustains an active plant root system, consequently delaying dormancy. Thirdly, a higher T_min_ may reduce radiative cooling effects, preventing the rapid cooling of the plant surface. This helps maintain a relatively high temperature at the plant surface, delaying the onset of dormancy [51]. Moreover, a reduced DTR may prompt plants to adjust their growth and dormancy schedules to accommodate relatively minor temperature changes, potentially resulting in an earlier onset of dormancy. Overall, the relative contributions of these mechanisms will vary depending on plant types, geographical locations, and environmental conditions. Therefore, comprehending the specific mechanisms by which a decrease in DTR impacts on vegetation EOS requires the consideration of various factors.

Across the majority of regions, we identified a negative correlation between the EOS and preseason precipitation. This pattern could potentially be attributed to the specific composition of vegetation. Areas characterized by widespread woody plants often maintain sufficient soil moisture, yet excessive rainfall could hinder root respiration, triggering an earlier EOS [52]. Furthermore, elevated precipitation in high-latitude zones might reduce autumnal solar radiation and photoperiods, hastening abscisic acid accumulation and accelerating leaf senescence [53]. In contrast, many regions displayed a significant positive correlation between the EOS and preseason precipitation, a connection potentially rooted in precipitation’s constructive impact on mitigating drought stress. The escalation of spring biomass due to global warming could result in summer and autumn water scarcity, curbing plant photosynthesis and advancing the EOS [1,54]. Consequently, increased precipitation supports physiological functions like photosynthesis and nutrient absorption during the growth season, contributing to delayed leaf senescence—a phenomenon which is particularly evident in arid regions [42,55].

### 4.4. Limitations

Although we have elucidated the climatic drivers influencing vegetation photosynthetic phenology in the mid–high latitudes of the Northern Hemisphere over the past two decades, this study still contains uncertainties. For instance, our analysis was primarily focused on the partial correlations of mean temperature, diurnal temperature range, and precipitation with vegetation photosynthetic phenology. We did not consider additional climatic variables such as cloud cover, solar radiation, or snow melt patterns, nor variations in vegetation types or human interventions [1,25,42,44]. Since our study predominantly relied on existing global climate databases, integrating emerging microclimate datasets in future work could enhance the accuracy of simulations and predictions regarding the impact of regional climate change on vegetation phenology [56]. Additionally, factors like the resolution of remote sensing data and the length of phenological and climatic data time series will significantly influence our research outcomes and should be further refined in subsequent studies.

## 5. Conclusions

By analyzing long-term satellite-derived data on vegetation photosynthetic phenology and corresponding climate information across northern terrestrial ecosystems from 2001 to 2020, we examined the dynamics and responses of vegetation photosynthetic phenology to preseason climate factors. The results indicate an advancing trend in the SOS of approximately −0.15 days yr^−1^ (*p* < 0.05), whereas the EOS showed a delaying trend with a rate of 0.17 days yr^−1^ (*p* < 0.01). Combined, these trends resulted in a lengthening of the growing season by 0.32 days yr^−1^ (*p* < 0.01), impacting 70.9% of our study area, but particularly Eurasia and eastern North America. We also found that, in the majority of regions, there was a significant negative correlation between the SOS and both T_mean_ and DTR. Similarly, there were more regions where the SOS was positively correlated with preseason precipitation than regions where it was negatively correlated. Nevertheless, there were noticeable regional differences in how climate factors affected the EOS of vegetation. The relationship between temperature factors and the EOS shows a roughly equal distribution in the areas with a significant positive correlation and a significant negative correlation. However, regions exhibiting a significant negative correlation between precipitation and EOS are far more numerous than those showing a significant positive correlation. The findings of this study suggest that changes in preseason DTR and precipitation have a notable impact on vegetation photosynthetic phenology. This presents a novel ecological indicator that aids in modeling and predicting the substantial influence of vegetation phenology on preseason climate regimes.

## Data Availability

The data used in the present work have been listed in the Data Sources.

## Supplementary Materials

The following supporting information can be downloaded at: https://www.mdpi.com/article/10.3390/plants13091254/s1, Figure S1: Spatial distributions of the preseason length for the partial correlation analysis of the start of the growing season and daily mean temperature, diurnal temperature range and accumulated precipitation based on phenology metric with 10% thresholds; Figure S2: Spatial distributions of the partial correlation coefficients between preseason environmental factors and the start of the growing season based on phenology metric with 10% thresholds; Figure S3: Spatial distributions of the preseason length for the partial correlation analysis of the end of the growing season and daily mean temperature, diurnal temperature range and accumulated precipitation based on phenology metric with 10% thresholds; Figure S4: Spatial distributions of the partial correlation coefficients between preseason environmental factors and the end of the growing season based on phenology metric with 10% thresholds.
