# Supplementary material for: Impact of Preseason Climate Factors on Vegetation Photosynthetic Phenology in Mid–High Latitudes of the Northern Hemisphere
Source: Plants (Basel). 2024 Apr 30;13(9):1254. doi: 10.3390/plants13091254 (PMC11085198; doi:10.3390/plants13091254)
Supplement: Supplementary file 1 [file plants-13-01254-s001.zip › plants-2955432-supplementary.pdf]

Supporting Information for

Impact of preseason climate factors on the vegetation photosynthetic phenology in mid-high latitudes of Northern Hemisphere

Contents of this file

Fig. S1 to S4

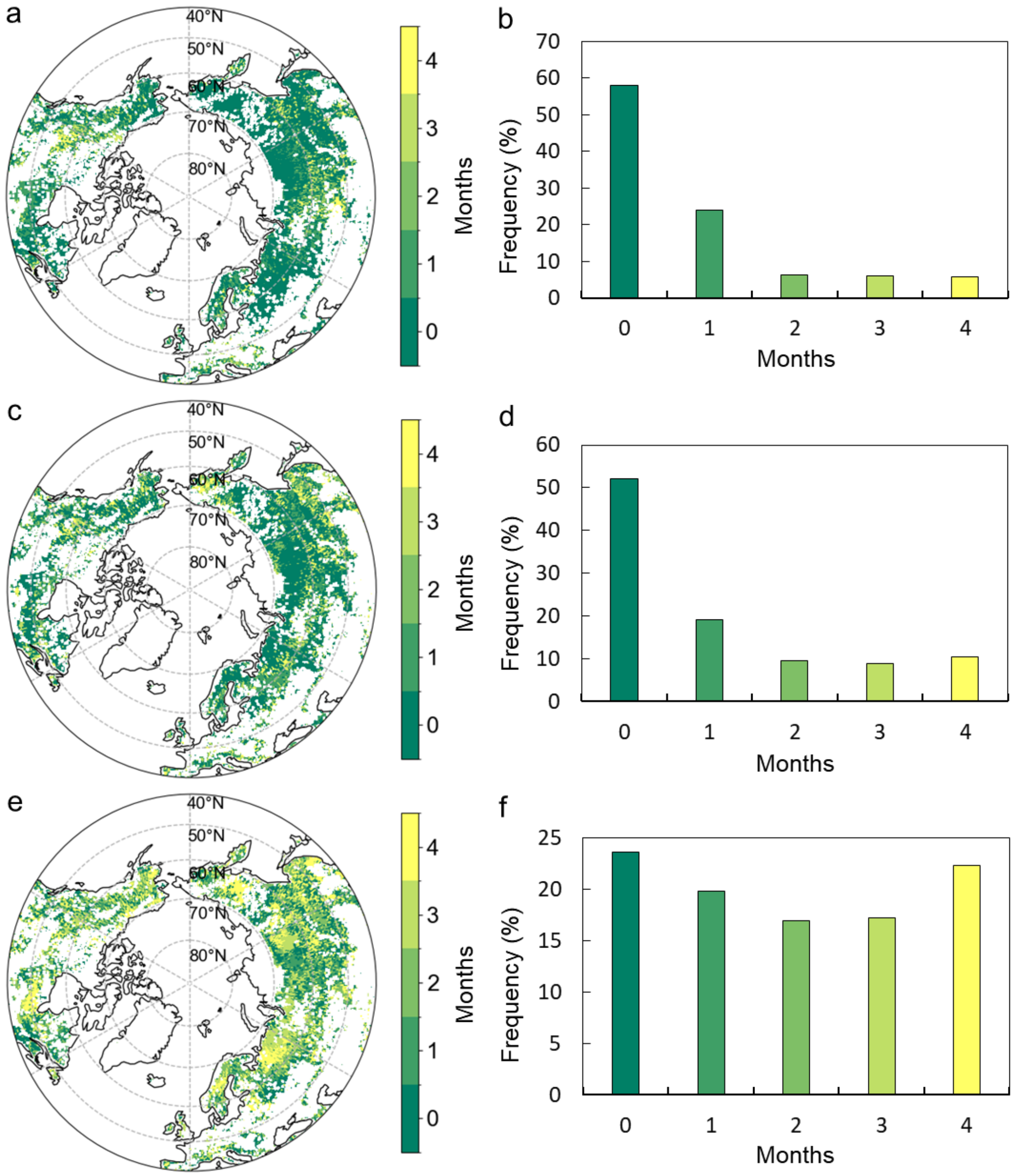

Fig. S1. Spatial distributions of the preseason length for the partial correlation analysis of the start of the growing season (SOS) and daily mean temperature (Tmean, A), diurnal temperature range (DTR, C) and accumulated precipitation (E) based on phenology metric with 10% thresholds. Uncolored pixels were excluded. Plots (B), (D) and (F) show the frequency distributions of preseason lengths in (A), (C) and (E), respectively.

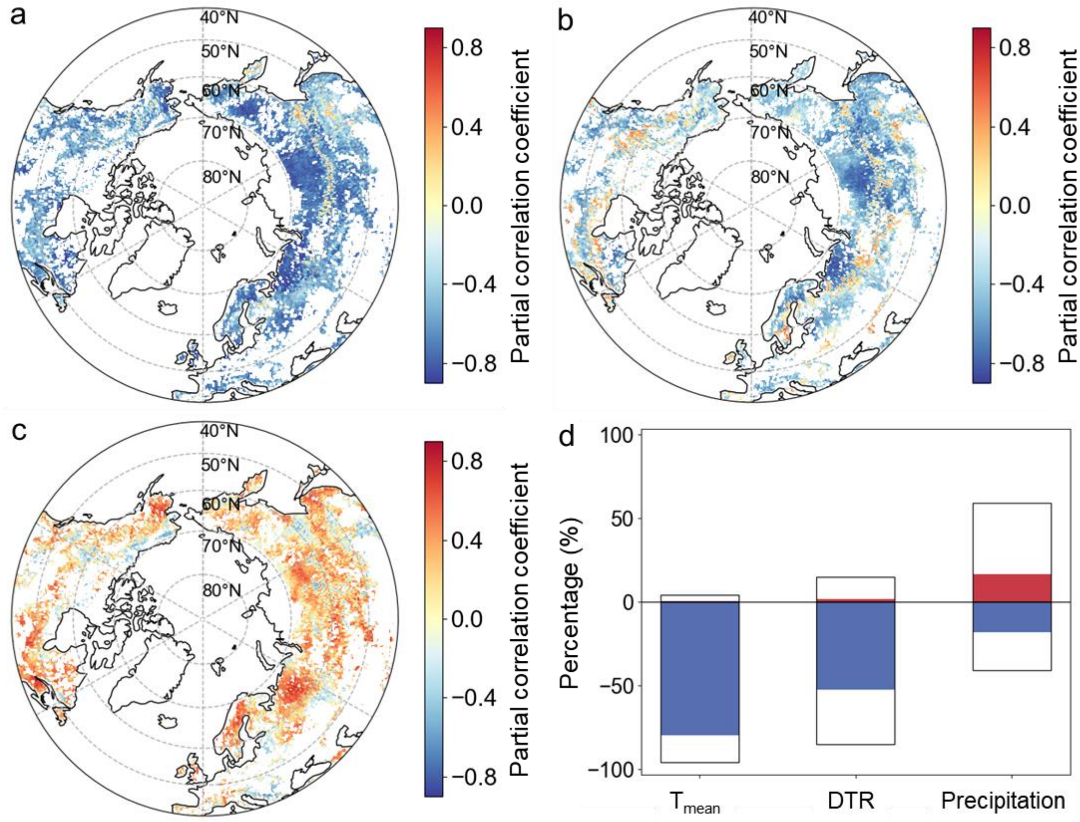

Fig. S2. Spatial distributions of the partial correlation coefficients between preseason environmental factors and the start of the growing season (SOS) based on phenology metric with 10% thresholds. (A) Spatial distribution of the partial correlation coefficient of daily mean temperature (T_{mean}) and SOS after controlling for accumulated precipitation. (B) Spatial distribution of the partial correlation coefficient of diurnal temperature range (DTR) and SOS after controlling for accumulated precipitation. (C) Spatial distribution of the partial correlation coefficient of accumulated precipitation and SOS after controlling for T_{mean}. Uncolored pixels were excluded. (D) Percentages of the partial correlation coefficients between environmental factors and SOS. The portion of the bars above the zero line indicates the percentage of positive correlation, while the portion below it indicates the percentage of negative correlation. The red and blue sections represent significant positive and negative correlations, respectively ($p < 0.05$).

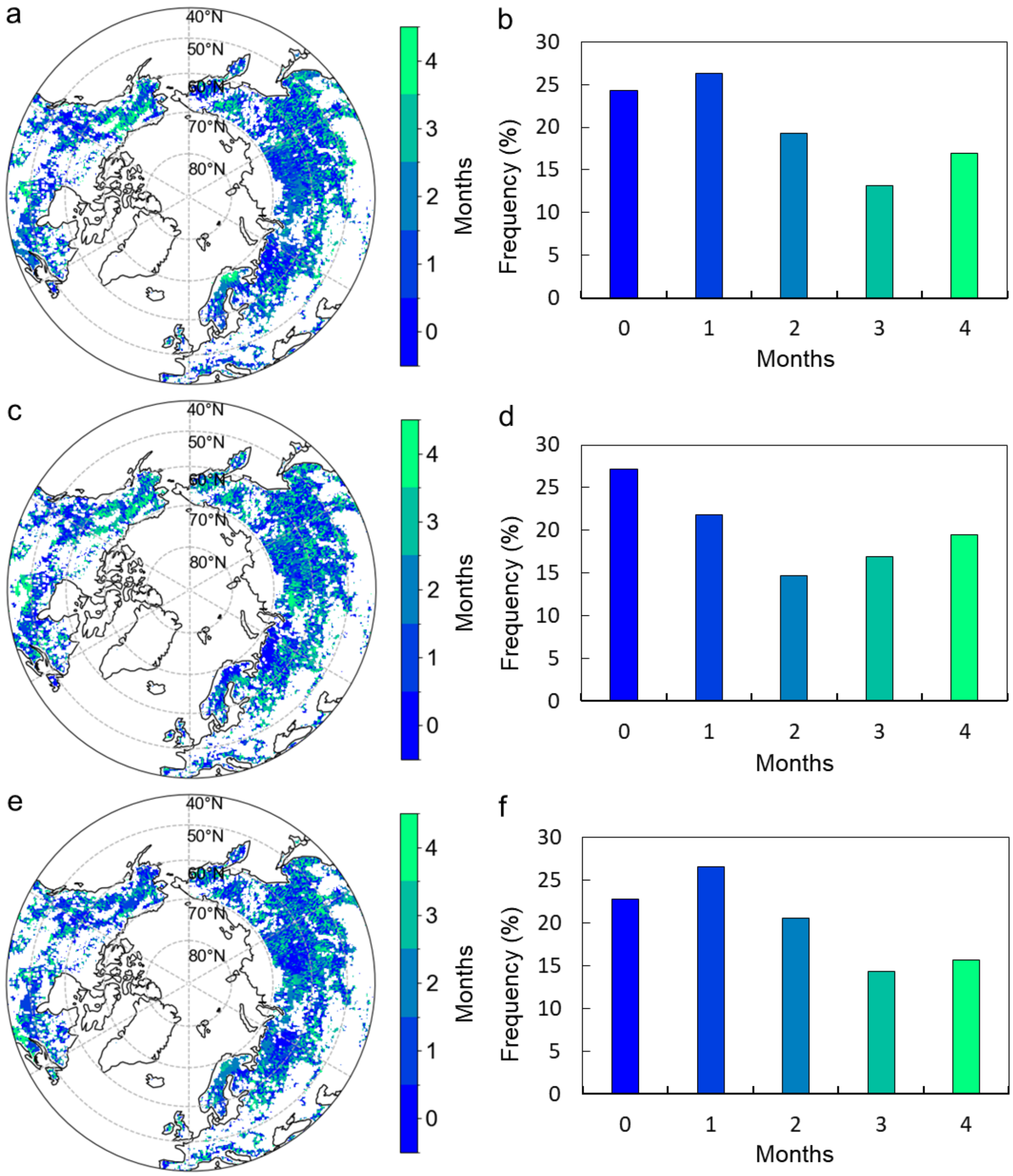

Fig. S3. Spatial distributions of the preseason length for the partial correlation analysis of the end of the growing season (EOS) and daily mean temperature (T_{mean}, A), diurnal temperature range (DTR, C) and accumulated precipitation (E) based on phenology metric with 10% thresholds. Uncolored pixels were excluded. Plots (B), (D) and (F) show the frequency distributions of preseason lengths in (A), (C) and (E), respectively.

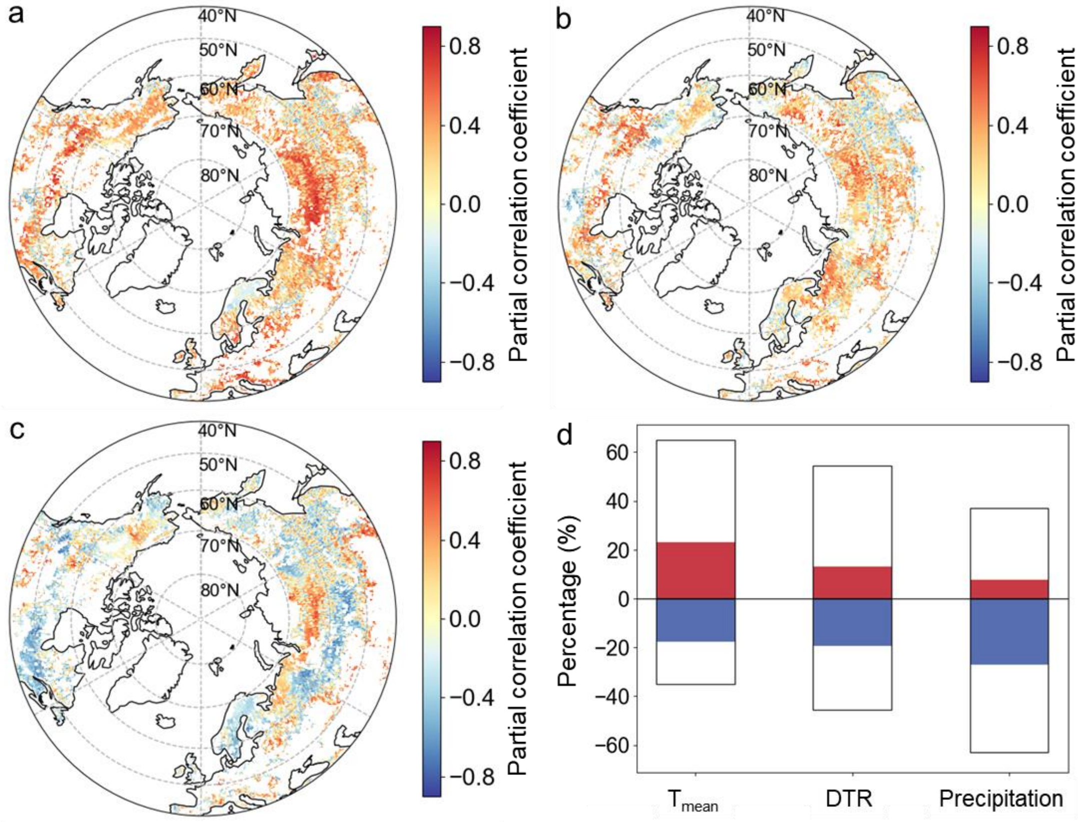

Fig. S4. Spatial distributions of the partial correlation coefficients between preseason environmental factors and the end of the growing season (EOS) based on phenology metric with 10% thresholds. (A) Spatial distribution of the partial correlation coefficient of daily mean temperature (T_{mean}) and EOS after controlling for accumulated precipitation. (B) Spatial distribution of the partial correlation coefficient of diurnal temperature range (DTR) and EOS after controlling for accumulated precipitation. (C) Spatial distribution of the partial correlation coefficient of accumulated precipitation and EOS after controlling for T_{mean}. Uncolored pixels were excluded. (D) Percentages of the partial correlation coefficients between environmental factors and EOS. The part of the bars above the zero line indicates the percentage of positive correlation, while that below it indicates the percentage of negative correlation. The red and blue sections represent significant positive and negative correlations, respectively ($p < 0.05$).
